# Electrophoresis of proteins and DNA on horizontal sodium dodecyl sulfate polyacrylamide gels

**DOI:** 10.1186/1742-4933-3-7

**Published:** 2006-07-12

**Authors:** Vincenzo Izzo, Maria A Costa, Renata Di Fiore, Giovanni Duro, Daniele Bellavia, Eleonora Cascone, Paolo Colombo, Maria C Gioviale, Rainer Barbieri

**Affiliations:** 1 Istituto di Biomedicina e Immunologia Molecolare, C.N.R., Via U. La Malfa, 153, 90153 Palermo, Italy; 2 Dipartimento di Biologia Cellulare e dello Sviluppo – Università di Palermo, V.le delle Scienze, 90128 Palermo, Italy; 3 Dipartimento GEN.UR.TO. – Università di Palermo, Italy

## Abstract

An inexpensive Plexiglas apparatus which allows a simple and rapid preparation of horizontal polyacrylamide gels of different dimensions for different purposes, is described. Preparation of such gels is as easy and rapid as agarose gel preparation, and polymerized polyacrylamide gels are used to fractionate proteins or small DNA fragments using a common horizontal electrophoretic tank. This apparatus was used to electrophoretically fractionate proteins or DNA for immuno-blot analyses, particularirly in the study of the allergenic response to *Parietaria judaica *pollen in senescence, for Southern-blot hybridizations and in the study of DNA polymorphisms.

## Introduction

Horizontal submerged agarose gel electrophoresis of nucleic acids is a basic tool of modern molecular biology [1]. Pouring melted agarose on a horizontal gel tray, the rapidity of polymerization, and the simplicity of handling and using polymerized agarose gels, are among the many advantages of this universally used technique. Unfortunately, proteins and very short nucleic acids are too small to be efficiently fractionated through the wide-pore agarose molecular sieve. Accordingly, a tighter matrix such as polyacrylamide is used to electrophoretically fractionate such small molecules [2]. Sodium dodecyl sulfate polyacrylamide gel electrophoresis (SDS-PAGE) is routinely used to obtain fractionation of proteins on the basis of their molecular mass. Since a polyacrylamide solution does not polymerize in the presence of oxygen, its polymerization is obtained by loading the solution between two glass plates., placed vertically, and a plastic comb is inserted in the upper slot side to form the loading wells [2]. This procedure, which often needs a stacking gel on top of the polymerized polyacrylamide, is long and tedious compared to horizontal agarose gel preparation. Indeed, polyacrylamide polymerization set arrangement imply these gels to be run vertically, and vertical gels of different dimensions each need a purpose-built apparatus.

We developed an inexpensive device to obtain horizontal polyacrylamide gels of different dimensions. Preparation of such gels is as easy and rapid as agarose gel preparation, and polymerized polyacrylamide gels are used to fractionate proteins or small DNA fragments using a common horizontal electrophoretic tank.

## Materials and methods

**Figure 1:**
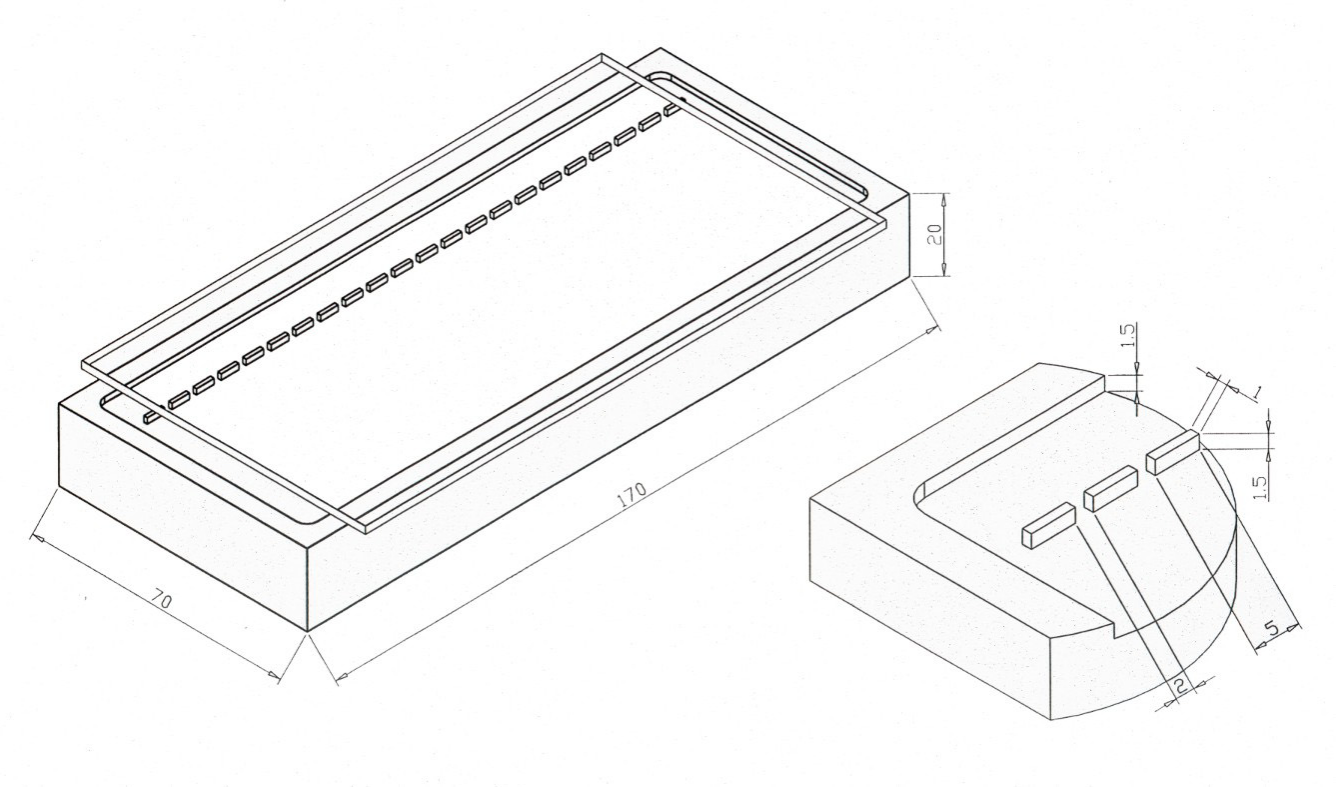
Schematic drawing of the apparatus A). Schematic section of the teeth and border frame B). The acrylamide solution was poured through one of the slots to fill the volume between the milled Plexiglas surface and the silanized glass plate.

A parallelepiped Plexiglas block (170 × 70 × 20 mm) was milled on one of its largest surfaces using a milling machine, to obtain a row of identical protruding teeth (5 mm × 1 mm × 1.5 mm) and a border frame 5 mm wide and 1.5 mm deep (Figure 1). A glass plate, 3 mm thick, was silanized on one surface with a silane solution (0.6% silane 96.4% ethanol – 3% acetic acid ; Silane from Sigma, USA). Silanization allows polymerized polyacrylamide to firmly adhere to the glass surface. The glass plate was then placed on the milled block, silanized surface down, to cover the milled surface, leaving a slot on both sides between the edges of the glass plate and the Plexiglas block border frame (Figure 1). A weight of about 100 g was placed on the plate to obtain a firm adhesion between the glass plate and both the protruding teeth and the border frame of the Plexiglas block.

A polyacrylamide solution, prepared by adding 15 μl of TEMED and 45 μl of 20% ammonium persulfate to 10 ml of acrylamide gel solution (Acrylamide/Bis 19:1, 10% w/v solution, 1 × TBE, pH 8.3, 0.1% SDS) was loaded through one of the slots to fill, by capillary action, the volume between the milled Plexiglas surface and the silanized glass plate. Under these conditions the polyacrylamide solution polymerizes in a few minutes. After polymerization, the glass plate with the gel firmly adhered to it, is removed from the device simply by inserting and rotating a scalpel between the glass plate and the Plexiglas border frame. The gel presents a row of wells with polyacrylamide walls and glass bottoms, and can be directly used in a common horizontal electrophoresis apparatus. The samples are loaded as they are in common horizontal agarose gels. H-SDS-PAGE was performed in 1X TBE, 0.1%SDS at 7 V/cm. We obtained gels of different dimensions and of different numbers of wells, either by positioning the glass plate differently on the milled surface of the Plexiglas block or using glass plates of different dimensions (see Figure 5).

If electrophoretically fractionated DNAs and proteins were to be transferred onto a membrane, the gel was removed from the glass support by passing a 0.4 mm nylon thread, tightly pressed with both hands on the glass surface, between the glass surface and the gel. Due to its consistency, the gel is easily "cut off" from the glass, preserving its integrity.

Electrophoretically fractionated DNA was stained by pouring glass-adhered gels in a 0.5 μg/ml ethidium bromide solution in 1X TBE and proteins were stained in a coomassie brilliant blue solution [1,3].

16HBE cell monolayers [4] were lysed in ice cold buffer (10 mM Tris, pH 7.4; 50 mM NaCl; 5 mM EDTA; 1% Nonidet P-40 and protease inhibitor cocktail (Boehringer Manheim) and centrifuged at 10000 g for 10 minutes. Supernatant protein concentration was determined by Bio-rad protein assay (Biorad, USA). Protein samples were denatured under reducing conditions, by boiling them for 5 minutes in sample buffer (50 mM Tris, pH6.8; 1% SDS; 2% B-mercaptoethanol; 0.01% bromophenol blue), before H-SDS-PAGE fractionation. Electrophoretically fractionated proteins were then electroblotted onto Immobilon-P membranes (Millipore, USA) which were then incubated overnight in blocking buffer (PBS 1X; 3% BSA; 0.5% Tween-20 and 0.02% NaN3). After three washes in PBS 1X; 0.5% Tween-20, the membranes were incubated for 1 hour at room temperature in the presence of mouse anti-HSP27 monoclonal antibody (Stressgen, clone G3.1) used at 1:1000 dilution. Detection of bound antibodies was carried out with the appropriate horseradish peroxidase-conjugated secondary antibody, followed by chemiluminescent detection using an ECL detection kit (Amersham, USA). Signal intensity was determined by densitometry (Biorad Gel doc 1000).

Western blot analysis of Parj2 recombinant protein, was performed as described elsewhere [5]

## Results

We performed electrophoretic fractionation of proteins or DNA using gels prepared with our apparatus in an electrophoretic tank commonly used for agarose gel electrophoresis.

### Electrophoresis of proteins

**Figure 2:**
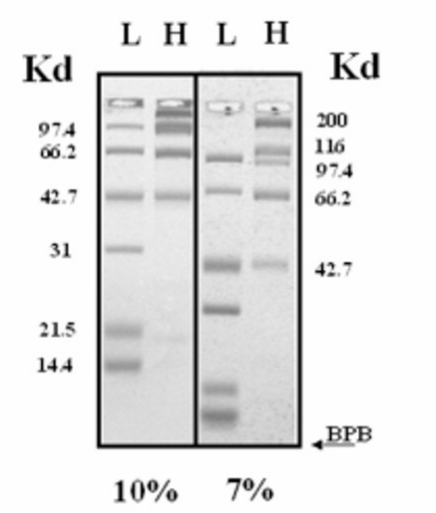
Low and high molecular mass marker proteins (Biorad, USA) were electrophoretically fractionated on a 10% (left) and 7% (right) H-SDS-PA gel, in 1X TBE, 0.1% SDS buffer until the dye ran off the gel. Gels were stained with coomassie brilliant blue as described in Materials and Methods.

In order to verify the linear relationship between the logarithm of molecular mass and the relative mobility of proteins, we have fractionated different protein markers (low and high molecular mass range standards, Biorad USA) on horizontal polyacrylamide gels of different concentrations (Figure 2). Electrophoretic migrations were carried out as described in Materials and Methods, until the bromophenol blue tracking dye ran off the gel. Using these parameters, 6 cm long gels and 45 minute runs, standard curves were generated by plotting the log of the molecular mass versus the migration distances. These curves are linear between 14.4–116 Kd (correlation coefficient r^2 ^= 0.985) on a 7% acrylamide gel, and between 14.4–66.2 Kd on a 10% acrylamide gel (r^2 ^= 0.990). High quality patterns of electrophoretic fractionation of proteins were obtained at both acrylamide concentrations used (Figure 2).

**Figure 3:**
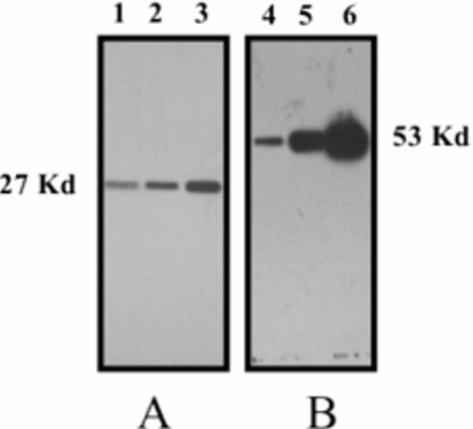
A) 1, 2 and 4 μg of 16HBE cell extracts (lanes 1, 2, and 3, respectively) were fractionated on a 10% H-SDS-PA gel and probed with mouse anti HSP27 monoclonal antibody as described in Materials and Methods. B) 0.5, 2.5 and 12.5 μg of Parj2 E. Coli lysate (lanes 4, 5, and 6, respectively) were fractionated on a 10% H-SDS-PA gel and probed with pooled allergic human sera, as described in Materials and Methods.

Western-blots were performed in the study of allergenic response to *Parietaria judaica *pollen in senescence, to demonstrate the efficiency and reliability of transfers using gels obtained with our device and recovered according to the procedure described in the Materials and Methods section. Figure 3A shows a western-blot analysis of 1, 2 and 4 μg (tracks 1, 2 and 3, respectively) of 16 HBE cell extracts (see Materials and Methods), probed with mouse anti HSP27 monoclonal antibody. Figure 3B shows a western-blot analysis of 0.5, 2.5 and 12.5 μg of Parj2 E. coli lysate probed with pooled allergic sera. Relative intensities of antibody binding signals indicates the reliability of western transfer.

### Electrophoresis of DNA

**Figure 4:**
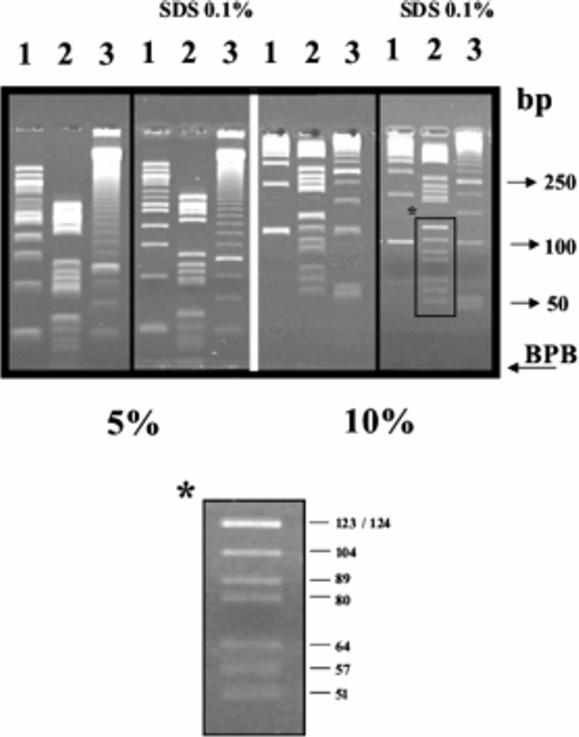
Different DNA molecular mass markers, 100 bp ladder (BioLabs, U.S.A.) (lane 1), Marker V (Roche, USA) (lane 2), 50 bp ladder (Amersham, USA) (lane 3), were electrophoretically fractionated on 5% or 10% polyacrylamide gel with or without SDS, until the dye ran off the gel. Gels were stained with ethidium bromide as described in Materials and Methods.

To electrophoretically fractionate small DNA molecules such as those obtained by polymerase chain reaction (PCR), polyacrylamide gels offer the highest resolution power. As polyacrylamide pore size is indipendent on the buffer used [6], electrophoretic fractionations were performed indifferently in TAE or TBE buffer. As the use of SDS-PAGE in DNA electrophoretic fractionation was suggested to enhance electrophoretic resolution power [7], we prepared horizontal polyacrylamide gels both in the presence or absence of SDS. As shown in Figure 4, DNA electrophoretic fractionation using 6 cm long gels in our apparatus, separates molecules which differ by only a few nucleotides.

**Figure 5:**
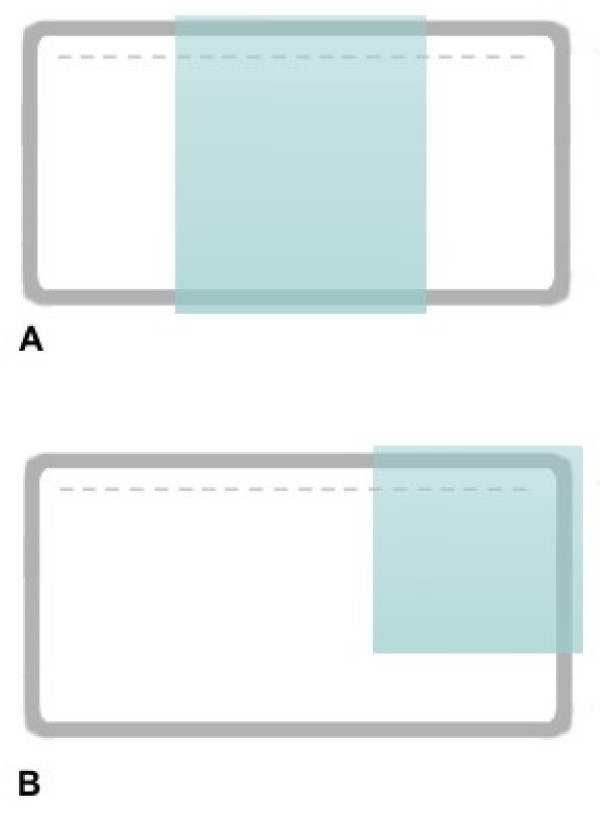
Plates of different breadth (A), and different positioning of the glass plate (B) allow to obtain gels of different number of wells and of different length, respectively.

H-SDS-PAGE fractionation patterns show a higher resolution power than the corresponding H-PAGE electrophoretic profiles (Figure 4).

## Discussion

We developed a simple, inexpensive and versatile device to prepare horizontal polyacrylamide gels for electrophoretic fractionation of proteins or DNA. The main advantages of our system are summarized as follows:

Preparation of H-SDS-PA gels is simpler and more rapid than conventional vertical gels.

Gels of variable length and breadth (number of wells) (Figure 5A and 5B respectively) can be obtained simply by using glass plates of different dimensions, or placing the glass plate differently, to cover the desired gel area on the milled Plexiglas surface. Furthermore, this avoids the need for separate electrophoretic apparata according to the desired gel dimension, as electrophoresis is performed on a common horizontal electrophoretic tank.

The tight adhesion between the gel and the glass plate offers a physical protection to the gel itself, so it can be easily handled. Indeed, the seal at the bottom of the wells, between the gel and the glass plate, has demonstrated to be absolutely reliable in hundreds of gels prepared with our device.

In DNA electrophoresis, a further advantage of gels prepared using our system is the possibility to check DNA band progression during fractionation, which is impossible when conventional vertical gels are used, in fact if necessary, after DNA staining and visualization as described in Materials and Methods, electrophoretic fractionation can be resumed.

A similar apparatus was built to obtain Microtiter Array Diagonal Gels (MADGE) for DNA genotyping analysis on polyacrylamide gels [7,8]. Although this method fully attains its aim by resolving short PCR products, it was applied only to fractionate DNA and for a specific purpose, in which both very short runs were performed and no particular gel resolution power was needed.
